# Nanomaterials Based on Honey and Propolis for Wound Healing—A Mini-Review

**DOI:** 10.3390/nano12244409

**Published:** 2022-12-10

**Authors:** Limberg Jaldin-Crespo, Nataly Silva, Jessica Martínez

**Affiliations:** 1 Regenerative Medicine Center, Faculty of Medicine, Clínica Alemana-Universidad del Desarrollo, Santiago 7610658, Chile; 2 Faculty of Design, Universidad del Desarrollo, Santiago 7610658, Chile

**Keywords:** wound healing, bionanomaterials, honey, propolis

## Abstract

Wound healing is a public health concern worldwide, particularly in chronic wounds due to delayed healing and susceptibility to bacterial infection. Nanomaterials are widely used in wound healing treatments due to their unique properties associated with their size and very large surface-area-to-volume ratio compared to the same material in bulk. The properties of nanomaterials can be expanded and improved upon with the addition of honey and propolis, due to the presence of bioactive molecules such as polyphenols, flavonoids, peptides, and enzymes. These bionanomaterials can act at different stages of wound healing and through different mechanisms, including anti-inflammatory, antimicrobial, antioxidant, collagen synthesis stimulation, cell proliferation, and angiogenic effects. Biomaterials, at the nanoscale, show new alternatives for wound therapy, allowing for targeted and continuous delivery of beekeeping products at the injection site, thus avoiding possible systemic adverse effects. Here, we summarize the most recent therapies for wound healing based on bionanomaterials assisted by honey and propolis, with a focus on in vitro and in vivo studies. We highlight the type, composition (honey, propolis, and polymeric scaffolds), biological, physicochemical/mechanical properties, potential applications and patents related of the last eight years. Furthermore, we discuss the challenges, advantages, disadvantages and stability of different bionanomaterials related to their clinical translation and insight into the investigation and development of new treatments for wound healing.

## 1. Introduction

Wound healing (WH) is a complex and dynamic biochemical and cellular process that takes place in four fundamental stages (hemostasis, inflammation, proliferation, and remodeling) [1,2,3]. The complete process takes a few weeks for acute wounds but could be extended to years for chronic wounds, and they have a significant negative impact on the wellness of patients and increase in the related healthcare costs [3]. Chronic wounds are characterized by a large inflammatory stage that blocks healing, increases neutrophil infiltration, promotes oxidative stress, and facilitates infections [4,5,6]. To address this health problem, researchers are making several efforts to develop novel therapeutic approaches to control infections and accelerate the chronic wound healing process.

Bionanomaterials are materials that have at least one of their dimensions on the nanometric scale and are synthesized by biological molecules [7,8]. Bionanomaterials tuned with natural products have demonstrated many advantages, such as cost-effectiveness, efficient delivery systems, and prevention of bacterial infections. They are non-toxic and biocompatible, non-scarring, sterile, biodegradable, and possess excellent mechanical and physicochemical properties [9,10,11,12]. Miguel et al., 2019, described examples of different bioactive molecules that have been loaded onto polymeric nanofibers, where the antibacterial biomolecules (e.g., antibiotics, silver nanoparticles and natural extract-derived products) and other molecules can enhance the healing process (e.g., growth factors, vitamins, and anti-inflammatory molecules). Among the principal advantages of nanofibers, we found: structural similarity with the skin extracellular matrix (ECM), high surface-area-to-volume ratio, porosity, capacity to act as a drug delivery system, support in cell adhesion, proliferation, and differentiation as well as act as a barrier for preventing the occurrence and establishment of infections. Furthermore, the addition of molecules (e.g., antibiotics, silver-based materials, molecules from natural extracts (e.g., essential oils, chitosan, Aloe vera, etc.), GFs, vitamins, and anti-inflammatory molecules) into electrospun membranes, as well as topical administration at wound sites, have been explored to avoid/impair skin infections and mediate the different phases of the healing process toward a more effective skin regeneration [10].

Andreu et al., 2015, analyzed different natural components (essential oils, honey, cationic peptides, aloe vera, plant extracts) as antimicrobial, anti-inflammatory and regenerative compounds to clarify their potential in clinical use as bioactive dressings. This study concluded that, for those natural occurring materials, more clinical trials are needed to corroborate their role as therapeutic agents in wound healing [11].

Vilches et al., 2020, reviewed and categorized forty-nine papers related to antioxidants loaded onto electrospun nanofibers to identify future applications and new trends. They found that inclusion of active compounds in nanofibers often improved the bioavailability of these compounds, increasing their stability, changing the mechanical properties of polymers, enhancing nanofiber biocompatibility, and offering new properties to required application [12].

Nour et al., 2021, performed a comprehensive overview of current and emerging angiogenesis induction methods applied in several studies for skin regeneration. Furthermore, Nour classified its methods in cell, growth factor, scaffold, and biological/chemical compound-based strategies. They concluded that the use of natural compounds that induced angiogenesis was not only economically appropriate, but due to their biocompatibility and presence of active molecules, the wound healing process was significantly accelerated. However, there might be sensitization and unpredictable side effects, since the mechanism of action for some of these compounds is still unclear, which is a major drawback. Currently, studies use a mixture of natural and synthetic materials to enhance the controllability of the properties of skin tissue-engineered constructs [13].

Adamu et al., 2021, reviewed the addition of bioactive ingredients, antibiotics drugs, anti-inflammatory agents, and traditional medicines into electrospinning solutions to produce new bioactive electrospun nanomaterials that might be released to the wound to enhanced the healing rate and provide antimicrobial properties to reduce infections. This review strongly argued that natural polymers and natural bioactive ingredients are leading in electrospun nanofibrous wound dressings, with numerous properties and advantages such as biocompatible, high swelling, non-toxic, antimicrobial, and cost-effective. In addition, they concluded that there has been no research conducted concerning comfort-related properties, and they are limited in their mechanical studies of electrospun nanofibers [14].

Lately, as an alternative to the conventional treatment of wounds, beekeeping products, such as honey and propolis, have emerged as a valuable tool, given their proven anti-inflammatory, antibacterial, antioxidant and wound healing promoting properties [11,12,15,16]. These properties of honey and propolis favor their addition to different polymers scaffolds such as chitosan [17], cellulose [18], poly (vinyl alcohol) (PVA) [19], polycaprolactone [20], gelatin [21] and polyurethane [22].

Hixon et al., 2019, and Bahari et al., 2022, highlighted multiple features (the antibacterial properties, low cost, biocompatibility and swelling index) of honey and honey-based nanoparticles for potential clinical use. Honey is often used without clinical modifications; there are also recent advances in wound healing with the use of honey-based nanoparticles [23]. This trend stimulates commercially available products ranging from dressings to gels. There is currently quite a large clinical application for honey, mainly used for the treatment of burns and ulcers. Unique nanotechnology and tissue-engineered scaffolds provide a novel delivery method for honey to the wound site, and several studies have explored their in vivo and in vitro uses [24]. Similarly, Bonsignore et al., 2021, and Tashkandi, 2021, agreed with the benefits for wound healing of different types of honey and remarked that there should be some type of standardization method to ensure equal bioactivity in every use [25,26].

Stojko et al., 2021, reviewed the potential use of biodegradable polymer nonwovens that release propolis as wound healing dressings. They concluded that the development of a biocompatible and biodegradable polymer dressing that releases propolis would significantly contribute to the effectiveness of wound treatment, as well as improve the patient’s quality of life [27]. Salama and El-Sakhawy, 2021, reviewed various composites prepared from propolis with polysaccharides such as cellulose, chitosan, starch, and alginate, where the chemistry, synthesis, and application are seriously discussed. This study found that a polysaccharide composite matrix with propolis may provide an appropriate platform for different applications such as wound healing. Moreover, enrichment of polysaccharides’ wound dressings with propolis will significantly improve their potential efficacy as wound dressing material. In addition, electrospun mats produced from cellulose-based composites can have a prospective application, due to their sustained release of active propolis from the nanofiber mat in wound healing mats [28].

Despite all these advances made, there are still no commercial bionanomaterials, for example electrospun gelatin-based nanofibers, available clinically for wound dressings. Recently, Li, Sun and Wu proposed three reasons. Firstly, the low productivity, poor reproducibility and a lack of standard operational methods and procedures for electrospun gelatin-based nanofibers severely hold back their commercialization. Secondly, although many existing studies have demonstrated that the integration of drug therapy and electrospun gelatin-based nanofiber mats are beneficial for wound healing, the best composition, concentration and release period remain unknown. Furthermore, the mechanism by which the different bioactive components promote wound healing remains unclear. Thirdly, the as-reported, improved wound healing efficiency by using drug electrospun gelatin-based nanofiber dressings was reported in rodent models, which have different regenerative capacity and mechanisms than humans [29].

**Figure 1:**
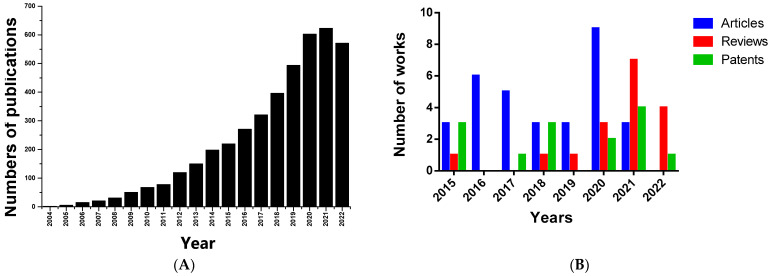
(**A**) Number of publications with the keywords “nanomaterials for wound healing” available in PubMed from 2004 to 2022 (obtained on 8 November 2022). (**B**) Number of publications (articles and reviews) and patents with the keywords “honey or propolis + nano + wound healing + scaffold” available in scientific publications and patent finders (WIPO and Espacenet) from 2015 to 2022 (obtained on 8 November 2022).

Over the past decade, the number of publications associated with key words “nanomaterials for wound healing” in PubMed has increased over the years (Figure 1A).

In order to make an original contribution in the field with this article, we have considered it relevant to focus on the last eight years of advances in the development of bionanomaterials, based on a wide variety of polymeric scaffolds available in combination with honey or propolis, and to focus on in vitro and in vivo studies. Literature on wound healing and bionanomaterials based on honey or propolis was searched for in the science databases PubMed, Scopus, and Web of Science and patent finders (WIPO and Espacenet). The search terms were “honey or propolis + scaffold + nanomaterial + wound healing” (Figure 1B).

This review focused on gathering the most recent advances in the development of bionanomaterials based on a wide variety of polymeric scaffolds available in combination with honey or propolis, focusing on in vitro and in vivo studies. We highlight the type of bionanomaterial, components composition (honey, propolis, and polymeric scaffolds), and associated properties assed to support their potential use in wound healing. We expect to provide new horizons on the potential and challenges related to their clinical translation. Finally, we discuss the pros and cons of these natural products combined with different polymeric scaffolds to provide insight into the research and development of new treatments in wound healing.

## 2. Wound Healing

**Figure 2:**
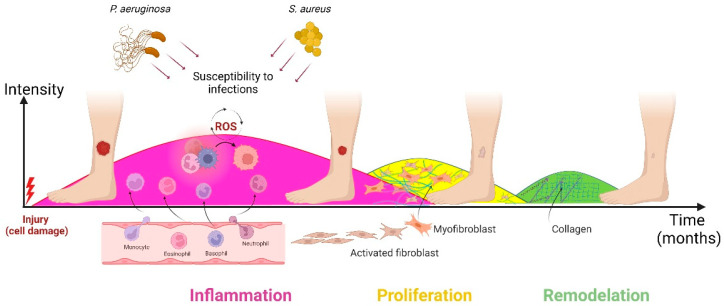
Healing stages of chronic wounds. The migration of phagocytic neutrophils and macrophages to the wound site initiates the inflammatory phase and leads to the release of more cytokines [35]. Oxidative environment due to the continuous production of reactive oxygen species (ROS) and prolonged pro-inflammatory phase promotes infections, and hypertrophic scars with collagen fibers in disorderly arrangement [36,37]. Created with BioRender.com.

Wound healing has four stages, but here, we describe bionanomaterials applications on three of them: inflammation, proliferation and remodeling, a period that ranges from the injury or trauma to the closure and complete healing of the wound in a time of days (acute wounds) or months (chronic wounds) (Figure 2) [30]. In the inflammatory stage, the action of lymphocytes, neutrophils, macrophages and platelets is highlighted, accompanied by the secretion of proinflammatory factors; in the proliferation stage, extracellular matrix synthesis, fibroplasia, angiogenesis, and re-epithelialization take place by fibroblasts, keratinocytes, and endothelial cells [31]. Finally, in the remodeling stage, extracellular matrix synthesis initiates wound closure and contraction, by the action of myofibroblasts [32].

Chronic wounds are increasing as a result of a rise in the prevalence of chronic diseases such as diabetes, cancer and cardiovascular alterations [33]. An unhealed wound on a foot or leg can lead to amputation. Furthermore, chronic wounds have a microenvironmental imbalance, particularly those that are infected [34].

## 3. Honey and Propolis Properties

**Figure 3:**
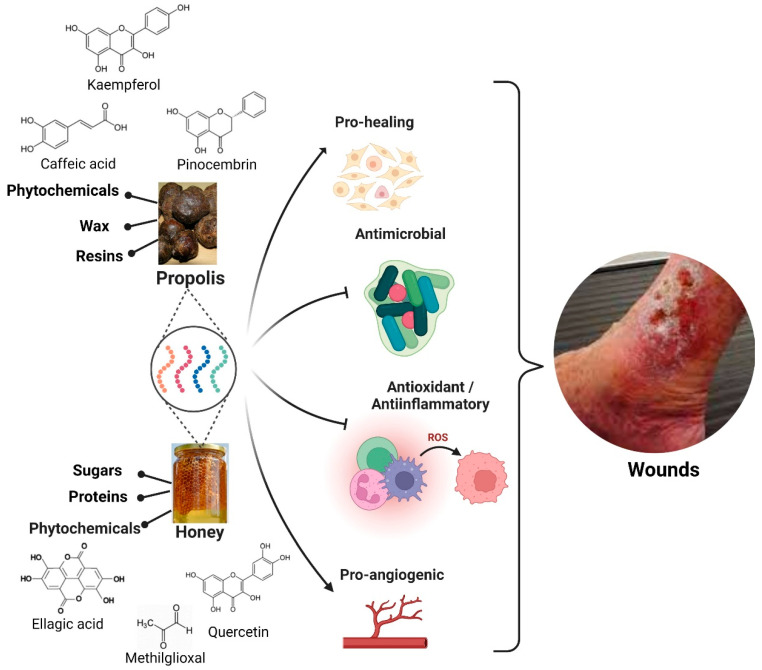
Properties of honey and propolis described for wound healing applications [6,39,40]. Created with BioRender.com.

Since ancient times, honey and propolis have been used for different purposes in medicine [38], particularly highlighting their effect as pro-healing, antimicrobial, antioxidant, anti-inflammatory and pro-angiogenic [6,39,40] (Figure 3).

### 3.1. Honey

Honey is an oversaturated solution of carbohydrates resulting from the digestive process of nectar collected from flowers and stored in the cells of the hive. It is mainly composed of monosaccharides such as fructose and glucose as well as disaccharides such as maltose, sucrose, among others. Furthermore, it has enzymes such as amylase, peroxide oxidase, catalase, and acid phosphorylase; moreover, it contains amino acids, vitamins, and antioxidants [41]. However, honey constituents are rather variable and depend primarily on its floral source; seasonal and environmental factors can also affect its biological properties [42]. Although not all honey has the same antimicrobial efficacy, to date, no microbial resistance has been associated with the use of honey, highlighting their useful potential [43,44]. To obtain improved properties, honey has been blended with different scaffolds at the nanoscale [45,46]. Furthermore, the commercial use of honey bionanomaterials is suitable for treatment at any healing phase [44,46].

#### 3.1.1. Antimicrobial Property

Honey is a natural antibiotic, efficient against resistant microorganisms and bacterial biofilms [47]. The antibacterial property of honey is due to its high osmolality, acidity, and content of glucose oxidase [24]. In addition, honey has been found to be effective against bacteria in biofilm, which is defined as the presence of communities of microorganisms in the wound bed that threatens the physiological healing process as the bacteria become 1000 times more resistant [43]. In particular, manuka honey has the power to act against biofilms due to methylglyoxal, which regulates fibrinogen formation and prevents the formation of biofilm structures in the wound bed [44].

#### 3.1.2. Anti-Inflammatory Property

The use of honey in wound treatments is suitable due to its anti-inflammatory activity and antioxidant capacity [48]. It eliminates free radicals, thus reducing prostaglandin levels, and acts as a vasoconstrictor, and therefore, inflammation, edema, and exudate in wounds decrease [10,49].

#### 3.1.3. Debriding Property

Honey contains several proteases that activate the plasminogen pathway, removing unhealthy tissue from a wound. These proteases become active due to the presence of hydrogen peroxidase in honey [50].

#### 3.1.4. Tissue Regenerative Property

Honey promotes epithelial growth, speeding up the healing process and tissue regeneration. In this regard, several clinical investigations found that honey can cure chronic ulcers in a short time [36].

### 3.2. Propolis

Propolis is a potent adhesive resinous substance, resulting from bee mastication of natural resins, which are collected from cracks in the bark and leaf shoots [37]. After adding salivary enzymes during chewing, bees add beeswax to enhance its final composition. Propolis contains resins (50%), wax (30%), essential aromatic oils (10%), pollen (5%), and other substances (5%), although its composition varies significantly according to the season and flower origin [51]. Propolis use for wound healing is due to the following properties:

#### 3.2.1. Antimicrobial Properties

Antimicrobial properties of propolis against Gram(+) and Gram(−) bacteria, protozoa, fungi, and viruses are due to its flavonoid content (pinocembrin, galangin, and pinobanksin, among others) [43,52].

Some of the most common constituents found in propolis, mainly quercetin and naringenin, promote an increase in membrane permeability and decrease in membrane potential, reducing bacterial resistance to antibacterial agents [53]. Moreover, flavonoids present in propolis reduce bacterial motility due to RNA polymerase inhibition [49].

#### 3.2.2. Antioxidant and Anti-Inflammatory Properties

Propolis has shown to trap free radicals, a mechanism by which it exerts its antioxidant potential [34]. Propolis also showed anti-inflammatory effects against chronicle and sharp models of inflammation. Rossi et al. [54] stated that propolis prevents cyclooxygenase activity in a concentration-dependent manner due to the presence of carvacrol, a monoterpenoid that acts as a potent activator of the TRPV3 (Transient Receptor Potential Subtype V3) and TRPA1 (Transient Receptor Potential Subtype A1) ionic channels. These channels are capsaicin receptors that play a key role as a mediator of inflammatory pain [54].

#### 3.2.3. Wound Healing Property

Propolis improves the growth of skin cells and its ability of remodeling and stimulates re-epithelialization via collagen expression, regulation of ECM components, and expression of transforming growth factor-β (TGF-β) [55]. This process involves the migration, proliferation of epidermal cells and keratinocytes, fibroblasts adherence, and contraction of ECM [56].

## 4. Bionanomaterials Based on Honey and Propolis

**Figure 4:**
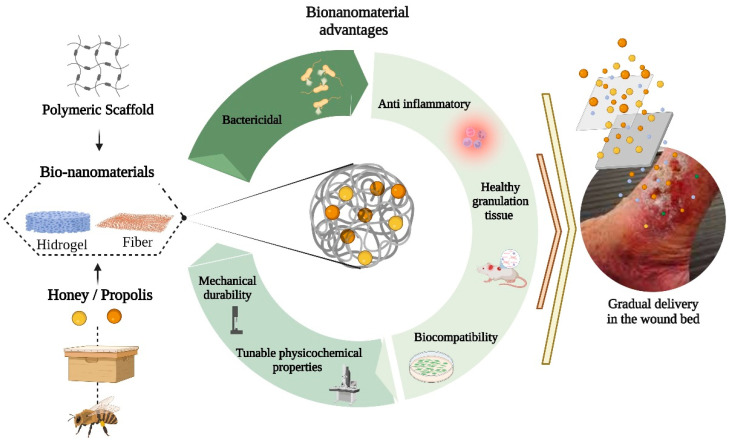
Advantages of bionanomaterials for wound healing. Honey and/or propolis blended with polymer scaffolds at the nanoscale improve the response rate of this therapy by modulating the wound microenvironment or by improving the penetration of multiple phytochemicals and other components of honey and propolis in the wound bed. Created with BioRender.com.

In recent years, enormous research efforts have been allocated to develop therapeutic systems for wound treatment. The bionanomaterials have demonstrated great performance for the effective treatment of wounds in vitro and in vivo [57,58,59]. The great surface area/volume ratio allows it to act as an active or passive carrier for delivering therapeutic agents [2,60,61,62]. In this way, the advantages of bionanomaterials based on honey and/or propolis blended with polymer scaffolds can improve their bactericidal, anti-inflammatory, and stimulation of healthy granulation tissue without decreasing their mechanical or physicochemical performance [62,63,64] (Figure 4).

Furthermore, bionanomaterials have also been reported to enhance their efficacy and reduce their negative effects in comparison with their macrosized counterpart or with the application of honey or propolis alone [28,62,64,65].

### 4.1. Polymeric Scaffolds

**Figure 5:**
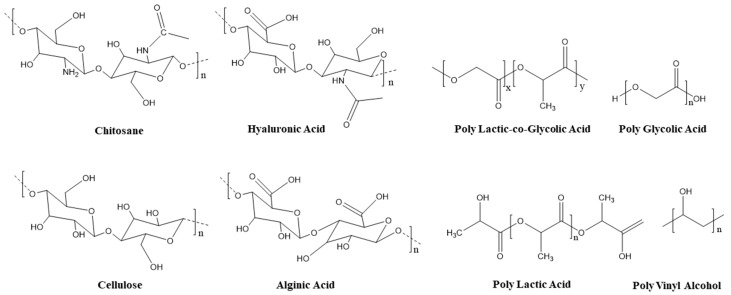
Polymeric scaffolds used in bionanomaterials applications. Natural polymers (chitosan, hyaluronic acid, cellulose and alginic acid) and synthetic polymers (poly lactic-co-glycolic acid, poly glycolic acid, poly lactic acid and poly vinyl alcohol) [32]. Both types of polymers are hydrophilic and absorb excess of wound exudates [73].

To promote wound healing, different polymeric scaffolds have been engineered to support cell adhesion, proliferation, differentiation, and orderly collagen deposition [66,67]. To achieve this goal, numerous efforts have been carried out to create dermal and epidermal substitutes to mimic human skin and controlled drug delivery systems [68]. Many natural and synthetic materials have been used as alternatives (Figure 5). However, the composition and architecture of the employed scaffolds affect their mechanical and physicochemical properties, which in turns affect the success of tissue regeneration [2,68,69]. Three different methods have been employed for the production of bionanomaterials scaffolds for wound healing: electrospinning, self-assembly, and phase separation [2].

In this context, an optimal bionanomaterial should release a specific therapeutic agent in the wound bed according to the wound healing stages. Drug delivery systems can be classified into passive, active, and smart systems [70], the addition of phytochemicals from natural products such as honey or propolis promotes an optimal microenvironment for wound healing. Passive approaches utilize a continuous release of drugs from the wound dressing [71]. On the other hand, active systems employ an external stimulus (e.g., temperature, electrical signal, light, etc.) for on-demand release [72]. Finally, smart systems detect the need for a specific drug based on diverse biomarkers produced in wound environments, which trigger the release of drugs when required [64].

One of the main goals of bionanomaterial as a drug delivery system is to control the release kinetics, based on carrier pore size, hydrophilicity, degradability, and its relative electrostatic charge compared to the drug. Jiao et al. showed that the mechanical properties of microfibers with a nanoscale topology hinder the transformation of fibroblasts into myofibroblasts on both random and aligned fibers, which is expected to prevent fibrosis [62]. Yun Xu et al. constructed an extracellular-matrix-mimicking nanofiber scaffold using silk fibroin and collagen and demonstrated potential on promoting fibroblast adhesion and growth [65]. Nanofibers and nanogels are the most widely used materials implemented as honey and propolis carriers in wound healing application [27,73,74,75,76]. Nanoscale of scaffolds and/or natural products have been utilized to better penetrate the wound bed and offer larger surface-area-to-volume ratio in comparison to their macroscopic counterparts [77].

### 4.2. Applications of Bionanomaterial Based on Honey for Wound Healing

Table 1 lists the publications about the use of honey-loaded bionanomaterials for topical use.

### 4.3. Bionanomaterial Based on Propolis for Wound Healing Applications

Table 2, lists publications about the use of propolis-loaded bionanomaterials for topical use.

### 4.4. Stability of Bionanomaterial Based on Honey or Propolis for Wound Healing

The stability of bionanomaterials is essential for their application in wound healing, for the different physicochemical, mechanical or biological parameters must be tested to ensure correct performance at the action site.

Swelling and dehydration are physicochemical features that can be used to determine the ability of wound dressings to absorb wound exudates, and this way can compare their behavior with and without the hive derivative. In regard to honey, several publications agreed that dissolution of nanofibers increases with higher honey concentration, due to an increase in solubility of the components present in honey [79,80,81,82,83,90]. The weight loss of bionanomaterials can be explained by the dual effect of leaching and increased hydrolysis [84]. For example, the presence of hydrophilic honey and Carica papaya in poliuretane (PU) enhanced the water absorption ability of the nanofiber samples to 761.67% from 285.13% in PU [75]. Bionanomaterials of PVA, chitosan, and honey enhance water uptake, but the swelling capability is moderate compared to spun chitosan and PVA fibers lacking honey. Honey increases the water uptake, leading to an increase in the degradation rate of fibers [74] M. A. Shahid et al. reported that enhancing the absorption rate of the PVA fiber in the presence of honey and turmeric extract decreases their intrinsic adhesive properties [83]. Additionally, S. Noori et al. demonstrated that the presence of honey affects the swelling of hydrogel nanocomposites, due to the presence of carboxylic acid groups in honey, which cause electrostatic repulsive forces and enhance swelling at pH 7 [67].

Regarding the thermal stability of the dressing, the results suggest that thermal degradation is susceptible to the addition of honey, regardless of the material in which it is incorporated. Honey acted as a good char insulator in the polymeric matrix. The presence of a crystalline structure and great compactness between the honey and Nepeta dschuparensis plant and PVA/Chit matrix led to high thermal stability of this bionanocomposite [79]. HPCS nanofibers with different honey concentrations exhibit good thermal stability below 120 °C [92] followed by a medium loss of 17% at 165–275 °C. Furthermore, the PU-HN-PA dressing also demonstrated a minor variation in degradation range, and at 500 °C, it lost ~86% of its total weight [75].

In relation to the thermal stability of propolis dressings, results agreed that the incorporation into PU matrices decreases orientation of the polymer chains and crystallinity [94,100]. However, the thermal stability of the as-formed cellulose acetate (CA)/propolis marginally increases when compared with CA nanofibers [97]. Similar results have been obtained in the bioAgNP–propolis-coated sutures, showing increased thermal stability compared with the noncoated silk sutures (control), with a mass change of around 51.16% from 250 to 465 °C [103].

Other physicochemical parameters have also been analyzed. For example, colloidal stabilization of nanoparticles/propolis for 30 days was analyzed by particle size, pH and Zeta potential. This study showed that nanoparticles remain stable, dispersed and with no aggregates [98]. PEG content increased solubility of propolis extract and provided greater stability in the formation of electrospinning fiber [99]. The chemical stability of the HPCS nanofibrous matrix with the apitherapeutics on the FTIR spectra did not show new chemical entities as a result of loading [82].

Tensile strength and elongation at maximum stress are two important mechanical properties that determine the stretchability of a wound dressing [66]. In general, results show an improvement in dressing elasticity due to the addition of honey [66,67,75,94] Z. Rafati et al. reported that tensile strength and elongation at maximum stress in groups treated with honey-loaded bionanocomposite hydrogel wound dressings were comparable, and in most cases higher, than the control group due to better collagenization [66]. The elastic behavior of poly(1,4-cyclohexane dimethylene isosorbide terephthalate (PICT)/honey nanofibers was improved by increasing the concentration of honey in the polymer solution, but this increase in concentration also caused a decrease in the Young’s modulus [76]. However, the presence of honey or S-nitroso-N-acetyl-penicillamine (SNAP) did not affect the stress/strain profiles or mechanical properties, suggesting that in this study, nanofibers have suitable elastic and tensile characteristics [91]. Furthermore, honey scaffolds of polycaprolactone had lower elastic moduli than the control without honey [85].

Finally, assessment of biological stability has been addressed to a lesser extent. R. Sarkar reported the in vitro stability profile of the PVA membranes and showed that membrane degradation was greater in PVA/honey nanofiber membranes [84]. On the other hand, A. Eskandarinia et al. demonstrated that a PU/propolis membrane was significantly resistant to both hydrolytic and enzymatic degradation [95].

### 4.5. Advantages and Disadvantages of Different Types of Bionanomaterials (Nanofibers and Nanogels)

**Figure 6:**
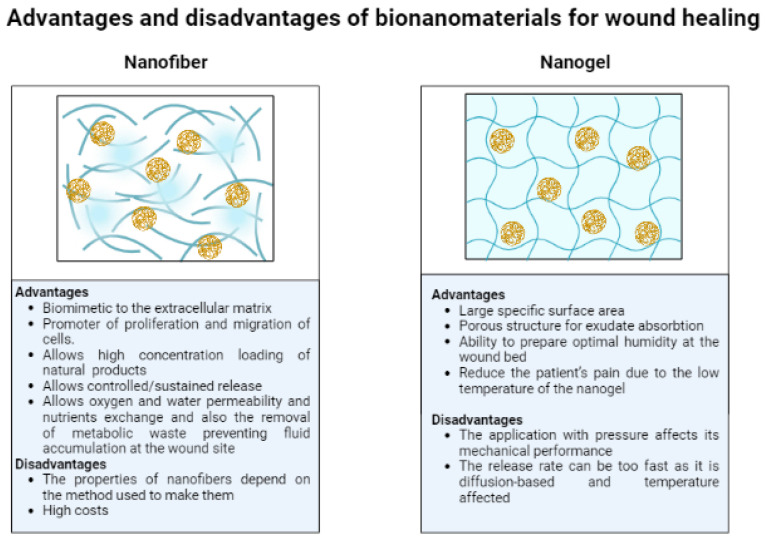
Advantages and disadvantages of the main bionanomaterials (nanofiber and nanogel) based on honey and propolis for wound healing [2,16,19,32,71,72,73,79,104,105,106]. Created by Biorender.com.

Numerous investigations have established the advantages and disadvantages of bionanomaterials based on honey or propolis for wound healing (Figure 6) [2,16,19,32,71,72,73,79,104,105,106].

### 4.6. Patents Related to Bionanomaterials Based on Honey or Propolis for Wound Healing

A search was performed in the international Patent Offices, “http://www.espacenet.com (accessed on 8 November 2022)”; “http://patentscope.wipo.int (accessed on 8 November 2022)”, for patents published from 2015 to 2022 using the terms “honey or propolis + nano + wound healing + scaffold”. This search yielded 14 patents, of which 13 used honey and 1 used propolis (Table 3).

## 5. Challenges, Future Directions, and Conclusions

Despite efforts made by researchers, there is limited success in the approval therapeutic use for bionanomaterials based on honey and propolis. This is due to the following reasons: (1) the complexity of the wound healing process, (2) unknown stability of new bionanomaterials, (3) limitations in the study models, mainly models of chronic wounds, (4) poor understanding of toxicity due to chronic exposure to new treatments, and (5) impact of these new treatments in the development of microbial resistance.

The new technologies act synergistically, stimulating the development, preparation, characterization, and in vitro/in vivo evaluation of bionanomaterials with a marked tendency toward using natural products such as honey and propolis. These bionanomaterials based on honey and propolis pursue ideal characteristics that act in each stage of both acute and chronic wounds. In the inflammatory phase, hive products act as free radical scavengers, inhibiting the production of proinflammatory factors and blocking the NFk-B pathway. In the angiogenesis stage, hive components contribute to revascularization, maintaining a favorable microenvironment for TGF-β synthesis and eventually modulating type II MMP synthesis to enhance collagen synthesis, which is a crucial step for re-epithelialization and final remodeling of the wound. Therefore, it is difficult to develop a bionanomaterial that is optimal for all stages or types of wounds.

The in vitro/in vivo models are dermal fibroblasts (murine/human), and the generation of wounds in a murine model. The limitations of these models generate barriers to a better clinical approach. For example, there have been different investigations that showed promising effects in vivo (animal models), but did not succeed in a clinical setting, probably due to the differences between wound microenvironments in humans and animal models. In this regard, more studies are required more complex models that resemble human skin. An ideal model of study to assess antimicrobial activity should consider a polymicrobial biofilm. To evaluate cytotoxicity, primary culture of fibroblasts derived from wounds or donor skin with chronic pathologies should ideally be used.

According to our findings in recently published studies, the most used polymeric scaffolds are polyvinyl alcohol, chitosan, and glycerol due to their high biocompatibility, low toxicity, and chemical characteristics that allow for the formation of non-covalent bonds with components derived from the hive. However, achieving a balance between physicochemical–mechanical–biological properties remain one of the first and big challenges in developing new biomaterials at the nanometric scale. Optimizing the rate between the bee products and the polymers affects the performance of the bionanomaterial for wound healing. For example, increasing the exudate absorption capacity of the bionanomaterial (by modulating the porosity or by changing the 3D structure of the scaffold) can affect the release kinetics of bioactive compounds loaded on the bionanomaterial, decrease the time of action around the wound bed, or module fibroblast signaling pathways. In the same way, a higher concentration of honey or propolis is expected to increase the antimicrobial and anti-inflammatory capabilities of the bionanomaterial. However, a non-optimal proportion of the scaffold and honey or propolis decrease the mechanical properties, such as their tensile strength, roughness or elasticity.

In this context, comprehensive studies are required to better understand the molecular mechanisms of new multilayer, biocompatible, biodegradable and biomimetic bionanomaterials loaded with natural products that enhance strategies with complementary mechanisms and that are ideally easy to apply in a real context. It is expected that these efforts using new technologies and evaluating effective models or tools pave the way for wound healing bionanomaterial therapy in the clinic.

## Figures and Tables

**Table 1:** Bionanomaterials based on honey. Type of bionanomaterial, composition, size, evaluation models, parameters tested (biological, physicochemical and mechanical), potential applications and references.

Type of Bionanomaterial	Composition	Size (nm)	Evaluation of Biological Parameters	Parameters Tested		Potential Applications	Ref.
				Biological	Physicochemical (PCP)		
					Mechanical (MP)		
Nanogel	Honey (10% *w*/*v*), PVA (60, 63.3, 66.7% *w*/*v*) dried egg-white (30, 31, 33.3% *w*/*v*), MMT (0.5, 10% *w*/*v*)	<100 nm	Female BALB/c mice	Wound closure for 10 days	PCP: Size, shape, swelling, water vapor permeability, thermal degradation, transparency, and honey release	Wounds with medium exudate and low microbial load	[66,69]
			Histological observations of healed wounds in BALB/c mice	Inflammation, cell proliferation, re-epithelization, angiogenesis, collagenization and tensile strength in healed skin	MP: Tensile strength and elongation at maximum stress		
			Human peripheral blood mononuclear cells	In vitro cytotoxicity assay			
Nanogel	Honey (15% *w*/*w*), PVA, (10% *w*/*w*), chitosan (2% *w*/*w*), MMT (0–3% *w*/*w*)	Undeclared	Total plate count method	Antibacterial (*S. aureus*)	PCP: Shape, chemical interaction, water vapor permeability, swelling and honey release	Wounds with medium exudate and medium microbial load	[67]
			Female Syrian mice	Wound closure for 12 days	MP: Tensile strength		
			Human peripheral blood mononuclear cells	Cytotoxicity			
Nanogel	Honey (5% *w*/*w*), PVA (94% *w*/*w*), borax (1% *w*/*w*)	<100 nm	Viable cell count method	Antibacterial (*S. aureus*, *E. coli*)	PCP: Size, shape, swelling, antibiotic release and bio-adhesion	Wounds with medium exudate and medium microbial load	[19]
			Human fibroblast cells	Proliferation	MP: Tensile strength an elongation at maximum stress		
				Cytotoxicity			
Nanogel	Honey (40% *v*/*v*), Nano-Zinc (20% *v*/*v*), nano-albumin (40% *v*/*v*)	<100 nm	Male albino mice	Wound closure for 10 days	NT	Third degree Burns	[78]
			Histological observations of the healed wounds in albino mice	Cell proliferation, angiogenesis and collagen synthesis			
Nanogel	Honey (~1.5% *w*/*w*), PVA (10% *w*/*w*), Chitosan (3% *w*/*w*), *Nepeta dschyparensis* (~1.5% *w*/*w*)	95–150 nm	Male Wistar rats	Wound closure for 21 days.	FCP: Size, shape, chemical interactions and thermal degradation	Second-degree Burns	[79]
			Histological observations of the healed wounds in Wistar rats	Cell proliferation, angiogenesis and collagen synthesis	MP: NT		
Nanogel	Honey (6% *w*/*v*), PVA (6% *w*/*v*), cellulose acetate (16% *w*/*v*), *Curcuma longa* extract (1% *w*/*v*)	262–695 nm	Disc diffusion assay	Antibacterial (*E. coli*)	PCP: Size, shape, chemical interactions, water vapor permeability and wettability	Wounds with medium exudate and medium microbial load	[80]
Nanofiber	Honey (0, 5, 10, 15, 20% *v*/*v*), PVA (7.2% *w*/*v*), Sodium alginate (0.8% *w*/*v*)	95–528 nm	Disc diffusion assay and dynamic contact assay	Antibacterial (*S. aureus* and *E. coli*)	PCP: Size, shape, chemical interactions, swelling, viscosity and conductivity.	Wounds with medium exudate and medium microbial load	[81]
			DPPH assay	Antioxidant capacity			
			NIH/3T3 cells	Cytotoxicity			
			DPPH assay	Antioxidant capacity			
Nanofiber	Manuka honey (10, 20, 25% *w*/*v*) and Lyophilized multiflora honey powder (10, 20, 25% *w*/*v*), Bee venom (0.01% *w*/*v*), PVA (9.7, 10.5, 12% *w*/*v*), extract of *Punica grantum* (1, 2, 2.5% *p*/*v*)	511–879 nm	Viable cell count method	Antibacterial (*S. aureus*, *E. coli*)	PCP: Size, shape and swelling	Wounds with medium exudate and medium microbial load	[44]
			Mouse fibroblast cells (L929)	Cytotoxicity			
			Female Sprague–Dawley rats	Wound closure for 14 days.	MP: NT		
Nanofiber	Honey (30% *w*/*v*), Propolis (10% *w*/*v*), bee venom (0.01% *w*/*v*), PVA (7% *w*/*v*), chitosan (3.1% *w*/*v*), bacteriophage (10% *v*/*v*)	319–997 nm	Viable cell count method	Antibacterial (*MRSA*, *P. aeruginosa*, *E. coli*),	PCP: Size, shape and chemical interactions	Infected chronic wounds with low to medium exudate	[82]
			Male mice	Wound closure for 12 days.			
			Histological observations of the healed wounds in albino mice	Necrosis, inflammation, collagen synthesis, vascularization and epithelialization			
			Human fibroblast cells	Cytotoxicity			
Nanofiber	Honey (33–50% *v*/*v*), PVA (5–6.7% *w*/*v*), *Curcuma longa* extract (0.03–0.06% *w*/*v*)	340 nm	Disc diffusion method	Antibacterial (*S. aureus*)	PCP: Size, shape, chemical interactions and wetness	Wounds with medium exudate	[83]
Nanofiber	Honey (0.2, 0.5, 1% *w*/*v*), PVA (12% *w*/*v*)	280–410 nm	Agar diffusion test, surface staining	Antibacterial, antibiofilm (*E. coli*)	PCP: Size, shape, chemical interactions, swelling, stability, conductivity and viscosity.	Control infections and inflammation and promote regeneration of the wound bed	[84]
			DPPH assay	Antioxidant capacity	MP: Roughness		
			Vero cells (kidney epithelial cells), quantification of bromodeoxyouridine (BrdU), scratch assay and expression of pre-inflammatory cytokines by Vero cells.	Cytotoxicity, proliferation, migration and inflammation			
Nanofiber	Honey (10, 20, 30, 40% *w*/*v*), PVA (5, 7, 10% *w*/*v*), chitosan (1.5, 3.5, 4.5, 5.5% *w*/*v*)	~500 nm	Viable cell count method	Antibacterial (*S. aureus*, *E. coli*)	PCP: Size, shape, chemical interactions, viscosity and swelling.	Wounds with medium exudate and medium microbial load	[74]
			Primary skin fibroblast cells of neonatal mice	Cytotoxicity			
Nanofiber	Honey (4% *w*/*v*), PU (4% *w*/*v*), *Carica papaya* extract (4% *v*/*v*)	170–210 nm	Human blood samples of healthy adults	Hemocompatibility (hemolysis)	PCP: Size, shape, chemical interactions, thermal degradation, porosity and pore size distribution, wettability, swelling, thermal degradation and protein absorption	Burns	[75]
				Coagulation (PT and APTT)			
Nanofiber	Honey (10, 15, 20% *w*/*v*), PICT (10% *w*/*v*)	190–482 nm	NT	NT	PCP: Size, shape, chemical interactions, honey release and wetness	Active wound dressing	[76]
					MP: Tensile strength		
Nanofiber	Manuka honey (1, 5, 10, 20% *v*/*v*), PCL (15% *w*/*v*)	500–5000 nm	Fibroblasts (CRL-252)	Cell viability	PCP: Size, shape, swelling and thermal degradation	Promoting healing and clearing bacteria from wound environment	[85]
				Proliferation, infiltration, and migration in vitro	MP: Elasticity		
			Agar diffusion test	Antibacterial (*S. agalactiae*, *E. coli*)	PCP: Size, shape, water vapor permeability and honey release		
Nanofiber	Honey (30–70% *w*/*w*), PDDA (30, 60, 70% *w*/*v*)	40–180 nm	Viable cell count method	Antibacterial (*S. aureus*, *E. coli*, *P. aeruginosa*)	PCP: Size, shape, chemical interactions and solubility	Antibiotic wound dressing	[86]
Nanofiber	Manuka honey (10, 20, 30, 40% *v*/*v*), Chitosan (7–35% *w*/*v*) loaded on a nanocomposite membrane: glycerol (30% *v*/*v*), dextran (48% *v*/*v*), nanosoy protein (22% *v*/*v*)	Undeclared	Zone of inhibition test and colony count method	Antibacterial (*S. aureus and E. coli*),	PCP: Shape, water vapor permeability, wettability and honey release	Multipurpose wound care membranes	[87]
			BALB/c mice	Wound closure for 21 days			
			Histological observations of the healed wounds in albino mice	Inflammation, migration, proliferation, angiogenesis, collagen synthesis, re-epithelialization	MP: TS		
Nanofiber	Manuka honey (10, 30, 50, 70% *w*/*v*), silk fibroin (20% *w*/*v*), PEO (2% *w*/*v*)	484–2229 nm	BALB/c mice	Wound closure for 21 days	PCP: Size, shape and chemical interactions	Control infections and promote the regeneration of the wound bed	[88]
			Measuring the bacterial growth-inhibition halos and bactericidal kinetics	Antibacterial (*S. aureus*, *MRSA*, *P. aeruginosa*, *E. coli*)			
			Mouse fibroblast cell line (L929)	Cell viability			
Nanofiber	Honey (10, 20 or 30%)/polyvinyl alcohol (7%)/chitosan (3.5%) (HPCS)	84 ± 97, 371 ± 110 or 464 ± 185 nm	Broth dilution method	Antibacterial (*S. aureus*, *E. coli*)	PCP: Size, shape, porosity, crystallinity, thermal degradation, swelling and degradation rate.	Wound healing and tissue engineering	[89]
Nanofiber	Honey (1–4 mL to 50%)/PVA (8%)	Undeclared	Mouse fibroblast cell line (L929)	Cytotoxicity	PCP: shape, composition, chemical interaction, swelling, crystallinity, conductivity, in vitro releasing kinetics analysis	Fabricated Band-Aids	[90]
Nanofiber	PLA (12%)/honey (5,10,15%) and PLA (12%)/honey/SNAP (10%)	624.92 ± 137.69 nm	In vitro bacterial adhesion assay Mouse fibroblast cell line (3T3)	Antibacterial (*S. aureus*, *E. coli*) Cytotoxicity, cell adhesion, cell proliferation	PCP: Size, shape, chemical interaction, wettability, swelling, water vapor transmission rate, NO release measurements, in vitro honey release, exudate absorption	Wound healing and tissue engineering	[91]
					MP: Tensile strength		
Nanofiber	polyamide 6 (16%)/honey (20%) nanofiber mats with boric acid (0, 5, 10, 15 and 20%)	253–304 nm	Disk diffusion method	Antibacterial (*A. baumannii*, *E. coli*, *P. aeruginosa and S. aureus*)	PCP: Size, shape, chemical interaction, thermal analysis, wettability, in vitro honey release, exudate absorption.	Wound healing applications	[92]

PVA: polyvinyl alcohol, PDDA: poly dialyldimethylammonium chloride, PCL: poly(ε-caprolactone), MMT: montmorillonite (magnesium aluminum hydroxysilicate), PEO: polyethylene oxide, PU: polyurethane, PICT: poly (1,4-cyclohexanedimethylene isosorbide terephthalate), DPPH: 2,2-Diphenyl-1-picrylhydrazyl, MRSA: methicillin-resistant staphylococcus aureus, NIH/3T3: fibroblast cell line that was isolated from a mouse NIH/Swiss embryo. HPCS: honey/polyvinyl alcohol/chitosan. SNAP: S-nitroso-N-acetyl-penicillamine.

**Table 2:** Bionanomaterials based on propolis. Type of bionanomaterial, composition, size, evaluation models, parameters tested (biological, physicochemical and mechanical), potential applications and references.

Type of Bionanomaterial	Composition	Size (nm)	Evaluation of Biological Parameters	Parameters Tested		Potential Applications	Ref.
				Biological	Physicochemical (PCP)		
					Mechanical (MP)		
Nanogel	Propolis (0.15% *w*/*v*), Carbapol 934 (0.5% *w*/*v*), nanosilver (0.05% *w*/*v*), Gelucire (0.1% *w*/*v*)	10.6–52.7 nm	Cup plate and broth dilution method	Antibacterial (*S. aureus*)	PCP: Size, shape and chemical interaction	Second-degree skin burns	[93]
			Wistar rats	Wound closure for 18 days	MP: NT		
Nanogel	Propolis (0.01, 0.02% *w*/*v*), collagen (2% *w*/*v*), chitosan (0.01, 0.02% *w*/*v*)	120 nm	Agar diffusion method	Antibacterial (*S. aureus*, *E. coli*)	PCP: Size, shape and chemical interaction.	Cutaneous wound healing applications	[94]
					MP: Elongation at maximum stress		
Nanofiber	Propolis (0.5, 1, 2 *w/v* %), PU (10 *w/v* %), HA (10 *w/v* %), DTA (7 *w/v* %)	294, 325, 718 nm	Female Wistar rats	Wound closure for 21 days	PCP: Size, shape, chemical interaction, thermal degradation, wettability and propolis release	Wounds with medium exudate and low microbial load	[95]
			Disc-diffusion method	Antibacterial (*S. aureus*, *E. coli*)	MP: Tensile strength and elongation at maximum stress		
			Histological observations of the healed wounds in Wistar rats	Inflammation and collagen synthesis			
			L929 mouse fibroblast cells (ATCC)	In vitro cytotoxicity assay			
Nanofiber	Propolis (40% *v*/*v*), PVP (6, 8% *w*/*v*), glycerol (40% *v*/*v*), nanosilver (10, 20% *w*/*v*)	~450 nm	Agar diffusion method	Antibacterial (*S. aureus*, *S. epidermidis*, *E. faecalis*, *E. coli*, *P. aeruginosa*, *Proteus vulgaris*, *Bacillus subtilis*, *Bacillus cereus*), Antifungal (*C. albicans*)	PCP: Size, shape, chemical interaction, AgNP release and propolis release	Wound healing stimulation with low microbial load	[96]
					MP: NT		
Nanofiber	Propolis (10, 20, 30, 40% *v*/*v*), Cellulose acetate (12% *w*/*v*)	150–200 nm	Inhibition zone method	Antibacterial (*S. aureus and E. coli*)	PCP: Size, shape, chemical interactions, propolis release and thermal degradation	Wound healing and antibacterial action	[97]
					MP: NT		
Nanofiber	Brazilian red propolis (10–60% *w*/*v*), PCL (27–60% *w*/*v*), Poloxamer (13–46% *w*/*v*)	200–400 nm	Culture in biphasic medium of *Leishmania chagasi*	Antimicrobial (*Leishmania braziliensis*)	PCP: Size, shape, chemical interactions and thermal degradation	Chronic wounds	[98]
			DPPH assay	Antioxidant capacity	MP: NT		
Nanofiber	Propolis (1.25% *w*/*v*), PVA (10, 15, 20, 30% *w*/*v*), PEG (1, 2% *w*/*v*)	282–984 nm	Male Swiss mice induced to diabetes with a single dose (150 mg/kg) of streptozotocin	Wound closure for 7 days.	PCP: Size and shape	Chronic wounds	[99]
			Murine NIH/3T3 fibroblast cells	Cytotoxicity	MP: NT		
Nanofiber	Propolis (undeclared), cellulose acetate (8, 10, 12, 14% *w*/*w*), PCL (14% *w*/*v*)	50–400 nm	Minimum inhibitory concentration assay	Antibacterial (*S. aureus*, *S. epidermidis*, *P. aeruginosa*, *E. coli*)	PCP: Size, shape, chemical interactions, and wettability	Wound healing system	[100]
			DPPH assay	Antioxidant capacity			
Nanofiber	Propolis (0.5, 1, 2% *w*/*v*), PU (10% *w*/*w*), HA (10% *w*/*v*)	~718 nm	Female Wistar rats	Wound closure for 21 days	PCP: Size, shape, chemical interactions, swelling, wettability, thermal decomposition and propolis release	Effective wound dressing for biomedical applications	[101]
			Histological observations of the healed wounds in Wistar rats	Cell proliferation, angiogenesis and collagen synthesis			
			Disc-diffusion method	Antibacterial (*S. aureus*, *E. coli*)			
			DPPH assay	Antioxidant capacity	MP: Tensile strength		
			L929 fibroblast cells	Cytotoxicity, proliferation, migration and inflammation			
Nanofiber	Propolis (5, 10, 20, 40, 60% *w*/*w*), PVA (8% *w*/*v*)	85–329 nm	Broth microdilution method	Antibacterial (*S. aureus*, *E. coli*)	PCP: Size, shape, chemical interactions, swelling, viscosity, and propolis release.	High exudate wounds and infection	[102]
			Primary skin fibroblast cells of neonatal mice	Cytotoxicity			
Nanofiber	Propolis (undeclared %), nonabsorbable 4.0 silk sutures, nanosilver (undeclared %)	Undeclared	Murine NIH/3T3 fibroblast cells	Cytotoxicity	PCP: Shape and thermal degradation	Antibacterial biomaterial for wound healing	[103]
				Migration in vitro	MP: NT		
			Agar diffusion test	Antibacterial (*S. aureus*, *E. coli*)			

HA: hyaluronic acid, DTA: dodecyltrimethylammonium salt, PVP: polyvinylpyrrolidone, PEG: poly-ethylene glycol.

**Table 3:** Patents, publication date, applicant and description of bionanomaterial based on honey or propolis.

Patent N°	Publication Date	Applicant	Name
IN202241032202	10.06.2022	DR. K KULATHURAAN.	Antibacterial nanomembrane for wound dressing and healing
CN211485249	15.09.2020	TAIZHOU ROOSIN MEDICAL PRODUCT Co., Ltd.	Novel honey dressing
IN202014043490	21.05.2021	SMART PRODUCTS & SERVICES INC. (DBA ALOEVIVE)	A combination of a novel topical gel and oral supplements for healing diabetic foot and other wounds
US20150030688	29.01.2015	SAINT LOUIS UNIVERSITY	Honey and growth factor eluting scaffold for wound healing and tissue engineering
CN1082837271	17.07.2018	GUANGDONG UNIVERSITY OF TECHNOLOGY	Nano-fiber dressing and preparation method thereof
CN112442278	05.03.2021	YANCHENG POLYTECHNIC COLLEGE	Preparation method of biomedical multifunctional nanofiber membrane
CN108359140	03.08.2018	SHAANXI YANGLING SHAANXI SPECIALTY AGRICULTURAL DEVELOPMENT CO., LTD	Honey-containing nanosilver antibacterial film and preparation method thereof
CN104342775	11.02.2015	NATIONAL DONG HWA UNIVERSITY	Method for preparing composite nanofiber membrane with honey and natural materials on basis of environmentally friendly electrospinning technology
CN108283727	17.07.2018	GUANGDONG UNIVERSITY OF TECHNOLOGY	Nanofiber dressing and preparation method thereof
CN111333918	26.06.2020	TIANJIN UNIVERSITY OF SCIENCE & TECHNOLOGY	Preparation method of dialdehyde nanocellulose/manuka honey antibacterial composite film
CN106620652	10.05.2017	PAN WEIFANG	Nano-ion antibacterial tissue regeneration promoting care solution and preparation method thereof
WO/2015/183228	03.12.2015	DUYMUŞ, Ethem	Nanofiber cover for wounds with an additive containing natural antiseptic
CN112442278	05.03.2021	YANCHENG POLYTECHNIC COLLEGE	Preparation method of biomedical multifunctional nanofiber membrane
CN110664725	10.01.2020	BEIJING ZHONGMI TECHNOLOGY DEVELOPMENT CO., LTD.	Preparation method of emulsion containing nanopropolis extract and prepared emulsion

## Data Availability

Not applicable.
